# Metabolic engineering *Saccharomyces cerevisiae* for *de novo* biosynthesis of hydroxycinnamoyl glycerols

**DOI:** 10.1016/j.synbio.2025.07.005

**Published:** 2025-07-17

**Authors:** Haiyan Zou, Chuanguang Xiao, Shujuan Zhao

**Affiliations:** The SATCM Key Laboratory for New Resources & Quality Evaluation of Chinese Medicine, The MOE Key Laboratory for Standardization of Chinese Medicines and Shanghai Key Laboratory of Compound Chinese Medicines, Institute of Chinese Materia Medica, Shanghai University of Traditional Chinese Medicine, Shanghai, 201203, China

**Keywords:** Hydroxycinnamoyl glycerols, *Saccharomyces cerevisiae*, Metabolic engineering, 1-*O*-*p*-coumaroylglycerol

## Abstract

Hydroxycinnamoyl glycerols (HCGs) are a class of bioconjugates characterized by the esterification of hydroxycinnamic acids with glycerol. Owing to their diverse biological activities, HCGs are considered high-value materials with potential applications in the food, pharmaceutical, and cosmetics industries. In this study, a *de novo* biosynthetic pathway of HCGs was constructed by integrating a *p*-coumaric acid-producing module and a 4-coumarate:coenzyme A ligase - hydroxycinnamoyltransferase (4CL-HCT) module into the genome of *Saccharomyces cerevisiae*. Through optimization of the primary metabolic pathway, fine-tuning of the shikimate pathway, and strategic selection of integration sites, the upstream metabolic flux in the *S. cerevisiae* strain was engineering to enhance the availability of l-tyrosine and l-phenylalanine precursors, as well as acetyl-coenzyme A (acetyl-CoA) and adenosine triphosphate (ATP) supply. As a result, the final engineered *S. cerevisiae* strain produced 1-*O*-*p*-coumaroylglycerol (1-OPCG) at a titer of 8.49 ± 2.29 μg/L in shake-flask fermentation. The strategies employed in this study provide a robust foundation for the synthesis of other HCGs.

## Graphical abstract

**Figure:**
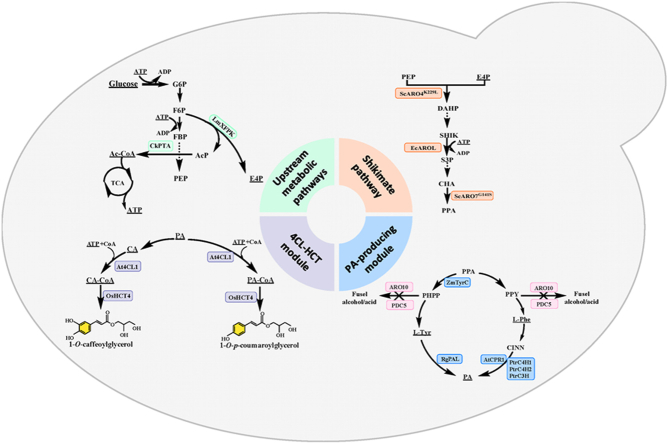

## 1 Introduction

Hydroxycinnamoyl glycerols (HCGs) are a class of compounds formed by the esterification of glycerol with hydroxycinnamic acids. Their basic chemical structure consists of a glycerol backbone (a three-carbon chain with three hydroxyl groups) to which a hydroxycinnamoyl moiety is attached. The hydroxycinnamoyl moiety typically consists of a hydroxyl and methoxy group decorated benzene ring (C6) linking with a C3 acrylic side chain [1]. This side chain is attached to the glycerol molecule by ester bonds, endowing these compounds a range of bioactive properties.

HCGs, such as 1-*O*-*p*-coumaroylglycerol (1-OPCG) and 1-*O*-caffeoylglycerol (1-OCG), exhibit significant biological activities such as neuroprotective and skin-protective effects, primarily attributed to its antioxidant properties, UV-filtering capacity, and favorable solubility characteristics [[1], [2], [3], [4], [5]]. These attributes them with broad application potential in the fields of food, pharmaceuticals, and cosmetics, positioning them as highly promising novel materials. At present, chemical and enzyme catalytic synthesis are the two main ways to synthesize HCGs [6,7]. Due to the limitations, such as lack of specificity and environmental burden of chemical synthesis [8], as well as limiting thermostability and narrow substrate scope of enzyme catalytic synthesis [9,10], synthetic biology-based microbial cell factory offers a powerful approach for the production of such valuable compounds.

**Fig. 1:**
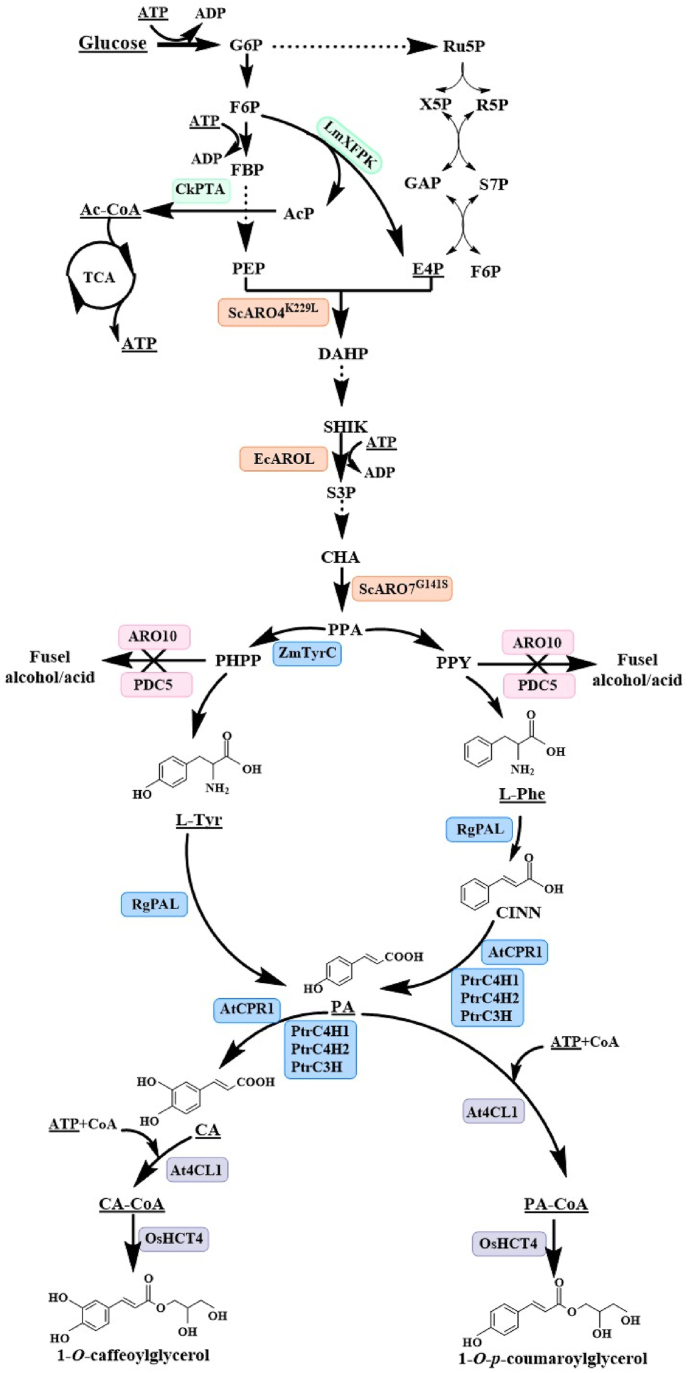
Engineering the biosynthesis of HCGs in yeast.

The hydroxycinnamoyl moieties in HCGs are predominantly derived from simple phenolic acids which are synthesized through the phenylpropanoid metabolic pathway, with l-phenylalanine (L-Phe) and l-tyrosine (L-Tyr) as the initial aromatic amino acid precursors. The production of PA in yeast can be accomplished via a single heterologous enzymatic step from L-Tyr or via two enzymatic steps from L-Phe [11]. L-Tyr is converted to PA by tyrosine ammonia lyase (TAL) [[12], [13], [14]] or phenylalanine ammonia lyase (PAL) [15,16]. By introducing a phenylalanine ammonia lyase (AtPAL2), a cinnamic acid hydroxylase (AtC4H), a cytochrome P450 reductase (AtATR2) from *Arabidopsis thaliana* and a cytochrome B5 from *Saccharomyces cerevisiae* (CYB5) [11], or a phenylalanine ammonia lyase from *Sorghum bicolor* (SbPal1), a cytochrome P450 reductase from *A. thaliana* (AtCpr1), and an identified Cyp complex (containing *Populus trichocarpa* cinnamic acid hydroxylase, PtrC4h2 and PtrC4h1, and coumarate-3-hydroxylase, PtrC3h3) [17], L-Phe can be converted to PA in the engineered microorganisms. In recent years, production of PA by engineering aromatic amino acid biosynthesis pathways has been achieved [11]. Additionally, PA was produced using cellulose or hemicellulose as the sole carbon source in engineered *Yarrowia lipolytica* [18]. These studies provide new approaches for efficient biosynthesis of PA. PA serves as a key intermediate and can be further modified through hydroxylation and methylation to generate various derivatives, including phenolic acids such as caffeic acid (CA) and ferulic acid (FA). In the presence of Adenosine triphosphate (ATP) and Coenzyme A (CoA), phenolic acids can then be catalyzed by 4-coumarate:coenzyme A ligase (4CL) to form hydroxycinnamoyl-CoA, such as *p*-coumaroyl-CoA (PA-CoA), caffeoyl-CoA (CA-CoA), and feruloyl-CoA, which are active molecules and acyl donors [19,20](Fig. 1).

Glycerol, the backbone part of HCGs, is a versatile and essential molecule that plays important roles in various aspects of life. The biosynthesis of glycerol proceeds chiefly through the glycolysis and glycerol-3-phosphate (G3P) pathways, starting with the reducing dihydroxyacetone phosphate (DHAP) to glycerol-3-phosphate (G3P) and then dephosphorylating G3P to yield glycerol successively catalyzed by NAD +-dependent glycerol-3-phosphate dehydrogenase and specific phosphatases.

The final step to form HCGs is catalyzed by hydroxycinnamoyltransferase (HCT), an enzyme that can utilize various acyl-CoA thioesters as acyl donors and catalyze the formation of esters or amides from a wide range of substrates, including shikimic acid, glycerol, malic acid, anthocyanins, spermidine, and quinic acid [[21], [22], [23], [24], [25], [26]]. Among them, OsHCT4 has the esterification ability by transferring the acetylated shikimic acid to glycerol [25]. Through co-expressing the 4CL from *Oryza sativa* (*Os4CL*) and *OsHCT4* in *Escherichia coli*, Kim et al. synthesized hydroxycinnamoyl-glyceride by biotransformation of PA, CA, and FA into corresponding glycerides, e.g. PA to 1-OPCG [25]. However, the *de novo* biosynthesis of 1-OPCG has not been reported.

During the biosynthesis of HCGs, ATP is required for multiple steps. In addition to the formation of hydroxycinnamoyl-CoA described above (Fig. 1), other processes such as the phosphorylation of glucose to generate glucose-6-phosphate (G6P), the phosphorylation of fructose-6-phosphate (F6P) to generate fructose-1,6-bisphosphate (FBP), and the phosphorylation of shikimate (SHIK) to generate shikimate-3-phosphate (S3P) also require ATP. The TCA cycle is a key pathway for ATP production in organisms [27], with acetyl-coenzyme A (acetyl-CoA) serving as a pivotal entry molecule [28].

As a generally recognized as safe (GRAS) microorganism, *S. cerevisiae* has many advantages for the production of natural products in food and pharmaceutical industrial. In this study, we firstly constructed a biosynthetic pathway of HCGs by integrating a PA-producing module and a 4CL-HCT module into *S. cerevisiae* genome. Combined engineering the *S. cerevisiae* with various strategies to improve the supply of L-Phe, L-Tyr precursors, and Adenosine triphosphate (ATP), *de novo* biosynthesis of 1-OPCG (Fig. 1) was achieved, providing the foundation for the biosynthesis of HCGs using yeast microbial cell factories. To the best of our knowledge, this is the first report of *de novo* biosynthesis of 1-OPCG in *S*. *cerevisiae*.

Solid arrows represent the confirmed reactions, while dashed arrows represent multiple-step reactions. The target molecules that were promoted through genetic engineering in this study are highlighted with underlines. **Metabolite abbreviations**: ATP, adenosine triphosphate; ADP, adenosine diphosphate; G6P, glucose-6-phosphate; F6P, fructose-6-phosphate; FBP: fructose-1,6-bisphosphate; AcP, acetyl-phosphate; Ac-CoA, acetyl-coenzyme A; TCA, tricarboxylic acid cycle, Ru5P, ribulose-5-phosphate; X5P, xylulose-5-phosphate; R5P, ribose5-phosphate; GAP, glyceraldehyde-3-phosphate; S7P, sedoheptulose-7-phosphate; E4P, erythrose 4-phosphate; PEP, phosphoenolpyruvate; DAHP, 3-deoxy-d-arabino-heptulosonate-7-phosphate; SHIK, shikimate; S3P, shikimate-3-phosphate; CHA, chorismate; PPA, prephenate; PHPP, 4-hydroxyphenyl pyruvic acid; PPY, phenylpyruvate; L-Tyr, l-tyrosine; L-Phe, l-phenylalanine; CINN, cinnamic acid; PA, *p*-coumaric acid; PA-CoA, *p*-coumaroyl-coenzyme A; CA, caffeic acid; CA-CoA, caffeoyl-coenzyme A. **Enzyme abbreviations**: LmXFPK, phosphoketolase from *Leuconostoc mesenteroides*; CkPTA, phosphotransacetylase from *Clostridium kluyveri*; ScARO4^K229L^, l-tyrosine-feedback-insensitive DAHP synthase from *S. cerevisiae*; EcAROL, shikimate kinase from *E.* *coli*; ScARO7^G141S^, l-tyrosine-feedback-insensitive chorismate mutase from *S. cerevisiae*; ARO10, phenylpyruvate decarboxylase, PDC5, pyruvate decarboxylase; ZmTyrC, feedback-insensitive cyclohexadienyl dehydrogenase from *Zymomonas mobilis*; RgPAL, phenylalanine ammonia lyase from *Rhodotorula glutinis*; AtCPR1, cytochrome P450 reductase from *A. thaliana*; PtrC3H3, coumarate-3-hydroxylase from *Populus trichocarpa*; PtrC4H1, cinnamic acid hydroxylase 1 from *P. trichocarpa*; PtrC4H2, cinnamic acid hydroxylase 2 from *P. trichocarpa*; At4CL1, 4-coumarate:coenzyme A ligase from *A. thaliana*; OsHCT4, hydroxycinnamoyltransferase from *Oryza sativa*.

## 2 Material & methods

### 2.1 Chemicals

L-Tyr, L-Phe, and PA were purchased from Yuanye (Shanghai, China), 1-OPCG was purchased from Yongjian (Jiangsu, China), and CA was purchased from BBI Life Sciences Corporation. These chemicals served as reference substances. When used in the substrate supplementary experiments, the chemicals were prepared in a stock solution in dimethyl sulfoxide (DMSO).

### 2.2 Microbial strains, vectors, and gene resources

*E. coli* strain DH5α was used for gene cloning. *S. cerevisiae* CEN.PK was used as host cell for recreating the HCG pathway. The shuttle expression plasmids pRS424 and pRS425 were used to construct gene expression vectors. All plasmids and microorganism strains used in this study are listed in Table 1, Table 2.

**Table 1:** Plasmids used in this study.

Plasmids	Features	Sources
pRS424	Amp, TRP1, 2μ ori, ori, FI ori	Our lab
pRS425	Amp, LEU2, 2μ ori, ori, FI ori	Our lab
pRS424-*LmXFPK*	pRS424; P_TEF2_-*LmXFPK*-T_TDH2_	This study
pRS424-*CKPTA*	pRS424; P_TDH3_-*CKPTA*-T_PGIT_	This study
pRS424-*ARO4*^*K229L*^	pRS424; P_TDH3_-*ARO4*^*K229L*^-T_ADH_	This study
pRS424-*ARO7*^*G141S*^	pRS424; P_TEF1_-*ARO7*^*G141S*^-T_PGIT_	This study
pRS424-*AROL*	pRS424; P_PGK1_-*AROL*-T_CYC1_	This study
pRS424-*RgPAL*	pRS424; P_PGK1_-*RgPAL*-T_TDH2_	This study
pRS424-*OsHCT4*	pRS424; P_TEF2_-*OsHCT4*-T_PDC1_	This study
pRS425-*At4CL1*	pRS425; P_TPI1_-*At4CL*-T_PGIT_	This study
pRS425-*Pc4CL1*	pRS425; P_TPI1_-*Pc4CL*-T_PGIT_	This study
pRS425-*Sm4CL2*	pRS425; P_TPI1_-*Sm4CL2*-T_PGIT_	This study

**Table 2:** Strains used in this study.

strains	description	Sources
*E. coli*	*E. coli DH5α F−, φ80d lacZΔM15, Δ(lacZYA-argF)U169, recA1, endA1, hsdR17(rk−, mk+), phoA, supE44λ−, thi−1, gyrA96, relA1*	In the lab
CEN.PK 2-1C	*S. cerevisiae* CEN.PK 2-1C, Mata; ura3-52, trp1-289, leu2-3,112,his3Δ1; MAL2-8c; SUC2	In the lab
HYC0	CEN.PK 2-1C, XVI:(P_TEF2_-*LmXFPK*-T_TDH2_)+(P_TDH3_- *CkPTA* -T_PGIT_) +(P_TDH3_- *ScARO4*^*K229L*^-T_ADH_)+(P_PGK1_- *EcAROL* -T_CYC1_)+(P_TEF1_- *ScARO7*^*G141S*^-T_PGIT_)+(P_URA3_-*URA3*-T_URA3_)	This study
HYC13	HYC0, XVI:(P_PGK1_- *RgPAL*-T_TDH2_)+(P_TRP1_- *TRP1*-T_TRP1_)	This study
HYC20	HYC13, XVI:(P_PGK1_-*ZmTyrC*-T_PGIT_)+ (P_TPI1_-*AtCPR1*-T_PDC1_)+(P_URA3_-*URA3*-T_URA3_)	This study
HYC21	HYC20, Δ*ARO10*::((P_TDH3_-*PtrC4H2*-T_FBA1_)+(P_PDC1_-*PtrC4H1*-T_CYC1_)+(P_TEF2_-*PtrC3H3*-T_FBA1_)+ (P_TRP1_-*TRP1*-T_TRP1_)	This study
HYC23	CEN.PK 2-1C, pRS424-*OsHCT4*, pRS425-*At4CL1*	This study
HYC24	CEN.PK 2-1C, pRS424-*OsHCT4*, pRS425-*Pc4CL1*	This study
HYC25	CEN.PK 2-1C, pRS424-*OsHCT4*, pRS425-*Sm4CL2*	This study
HYC26	HYC21, Δ*PDC5*::(P_TPI1_-*At4CL1*-T_PGIT_)+(P_TEF2_-*OsHCT4*-T_PDC1_)+(P_LEU2_-*LEU2*-T_LEU2_)	This study
HYC27	HYC21, Δ*PDC5*::(P_TEF2_-*At4CL1/(GGGGS)*_*3*_*/OsHCT4*-T_PDC1_)+(P_LEU2_-*LEU2*-T_LEU2_)	This study
HYC28	HYC21, Δ*PDC5*::(P_TEF2_-*OsHCT4/(GGGGS)*_*3*_*/At4CL1-*T_PDC1_)+(P_LEU2_-*LEU2*-T_LEU2_)	This study

Genes, including *RgPAL*, *ZmtyrC*, *AtCPR1*, *PtrC3H3*, *PtrC4H1*, *PtrC4H2*, and *OsHCT4* for recreating the 1-OPCG pathway, and *LmXFPK* and *CkPTA* for promoting the upstream metabolic flux, were codon-optimized for *S. cerevisiae* and synthesized by Sangon (Shanghai, China). The other genes, including *ScARO4*, *ScARO7*, and *EcAROL* for engineering the upstream metabolic pathways, and *Sm4CL2*, *Pc4CL1*, and *At4CL1*, for constructing the HCG pathway, were obtained through PCR amplification. *ScARO4*^*K229L*^ and *ScARO7*^*G141S*^ mutants were obtained by site-directed mutagenesis method according to literature [29]. The information of all these genes is shown in Table 3, and the primers used in this study are listed in Table S1.

**Table 3:** Genes used in this study.

Gene	Enzyme	Resource	GenBank ID/Gene ID
*LmXFPK*	Phosphoketolase	*Leuconostoc mesenteroides*	AY804190.1
CkPTA	Phosphotransacetylase	*Clostridium kluyveri*	WP_012101779.1
*RgPAL*	Phenylalanine ammonia lyase	*Rhodotorula glutinis*	AUQ35650.1
*OsHCT4*	Hydroxycinnamoyltransferase	*Oryza sativa*	115466807
*ZmtyrC*	Feedback-insensitive cyclohexadienyl dehydrogenase	*Zymomonas mobilis*	AAA27684.1
*AtCPR1*	Cytochrome P450 reductase	*Arabidopsis thaliana*	NM_118585.4
*PtrC3H3*	Coumarate-3-hydroxylase	*Populus trichocarpa*	EU603301.1
*PtrC4H1*	Cinnamic acid hydroxylase 1	*P*. *trichocarpa*	XM_002325602.3
*PtrC4H2*	Cinnamic acid hydroxylase 2	*P*. *trichocarpa*	XM_002319938.3
*Sm4CL2*	4-coumaric acid:coenzyme A ligase	*Salvia miltiorrhiza*	AY237164
*Pc4CL1*	4-coumaric acid:coenzyme A ligase	*Petroselinum crispum*	X13324
*At4CL1*	4-coumaric acid:coenzyme A ligase	*A*. *thaliana*	NM104046
*ScARO4*	DAHP synthase	*S*. *cerevisiae*	852551
*ScARO7*	Chorismate mutase	*S*. *cerevisiae*	856173
*EcAROL*	Shikimate kinase II	*E*. *coli*	945031

### 2.3 Expression vector construction and expression cassette amplification

All plasmids were constructed according to standard molecular cloning protocols. *E. coli* transformants were selected and maintained on Luria−Bertani (LB) plates containing 100 mg/L of ampicillin, and *S. cerevisiae* transformants were cultured in synthetic dropout (SD) selection media SD-Trp and SD-Leu for plasmids pRS424 and pRS425, respectively.

To construct the expression vector, the promoter regions of *PTEF2*, *PTDH3*, *TEF1*, *PPGK1*, *PTPI1*, and *PPDC1* and the terminator regions of *TTDH2*, *TPGIT*, *TADH*, *TCYC1*, *TFBA1*, and TPDC1 were firstly PCR amplified using gene-specific primers and genomic DNA of *S. cerevisiae* as template. Then, the promoter and terminator fragments were combined and inserted into pRS424, pRS425 or pRS426 to get the shuttle expression vectors pRS424-P_TEF2_-MCS-T_TDH2_, pRS424-P_TDH3_-MCS-T_PGIT_, pRS424-P_TDH3_-MCS-T_ADH_, pRS424-P_TEF1_-MCS-T_PGIT_, pRS424-P_PGK1_-MCS-T_CYC1_, pRS424-P_PGK1_-MCS-T_TDH2_, pRS424-P_TEF2_-MCS-T_PDC1_ and pRS425-P_TPI1_-MCS-T_PGIT_. Finally, *LmXFPK*, *CkPTA*, *ScARO4*^*K229L*^, *ScARO7*^*G141S*^, *EcAROL*, *RgPAL*, *OsHCT4* and 4CL were separately inserted into the multiple cloning site (MCS) of the above vectors.

Through homologous recombination, the 5′- and 3′- ends of *ZmTyrc*, *AtCPR1*, *PtrC3H3*, *PtrC4H1*, and *PtrC4H2* were respectively connected to the promoter and terminator regions, resulting in P_PGK1_-*ZmTyrc*-T_PGIT_, P_TPI1_-*AtCPR1*-T_PDC1_, P_TEF2_-*PtrC3H3*-T_FBA1_, P_PDC1_-*PtrC4H1*-T_CYC1_, and P_TDH3_-*PtrC4H2*-T_FBA1_.

To get an optimal HCT-4CL module, pRS424-*OsHCT4* combined with pRS425-*At4CL1*, pRS425-*Pc4CL1*, or pRS425-*Sm4CL2* were separately co-transformed into *S. cerevisiae* CEN.PK to generate yeast strains HYC23, HYC24, and HYC25, respectively (Table 2). In addition, two fusion proteins of At4CL1-OsHCT4 and OsHCT4-At4CL1 were also evaluated as the HCT-4CL module. The fusion proteins were constructed by overlap PCR using a flexible linker (GGGGS)_3_ by referring literatures [17,[30], [31], [32]] to connect *At4CL1* and *OsHCT4*.

The constructed expression vectors were used as templates to do PCR amplification to get the whole gene expression cassette of individual genes for targeted integration into the genome of *S. cerevisiae*.

### 2.4 Gene expression cassette integration into *S. cerevisiae* genome

Gene expression cassette targeted integration was conducted using homologous recombinant method as described [33]. Briefly, the up- and downstream 350–900 bp regions flanking the selected integration sites, including YPRC△15 on chromosome XVI [33], expression cassette of selection marker genes introduced on chromosome XVI, *ARO10* on chromosome IV, and *PDC5* on chromosome XII, and the corresponding gene expression cassette were amplified by PCR, using individual primers listed in Table S1. The PCR products were overlapped and spliced by SOE PCR, and then targeted integration of multiple gene cassettes was achieved through co-transformation, as shown in Fig. S1. Positive clones were verified by colony PCR and sequencing.

### 2.5 Shake flask fermentation and product extraction

For shake-flask culturing, seed cultures of engineered yeast were cultured by picking a single colony and grown in 5 mL of either SD selection medium or YPD medium at 30 °C, 220 rpm for 24 h. To evaluate the intracellular L-Tyr and L-Phe levels of the engineered strain, 5 % of seed cultures were inoculated into SD medium and cultivated under the same conditions for another 120 h. To extract the intracellular L-Tyr and L-Phe, cells were mixed with glass beads and phosphate buffer in a volume equivalent to that of the fermentation broth. The mixture was mixed well, kept in ice for 1 min; this process was repeated 5 times. The supernatant was then collected and filtered through a 0.22 μm filter for analysis.

Extracellular product content was used to evaluate enzyme-catalyzed activity. To do that, 5 % of the seed culture was inoculated into 20 mL SD selection medium and cultivated under the same conditions for another 24 h; then 25 g/L glycerol and 100 mg/L of PA or CA were added, if required, and cultured for another 72 h. For PA content determination, 5 % of the seed culture was inoculated into 20 mL YPD liquid medium (20 g/L of glucose, 20 g/L of peptone, 10 g/L of yeast extract) and incubated for 120 h under the same conditions. Then, 1 mL of fermentation broth was taken and centrifuged at 12000 rpm for 2 min. The supernatant was then filtered through 0.22 μm filter for analysis.

For 1-OPCG content determination, 5 % of the seed culture was inoculated into 20 mL YPDG liquid medium (YPD medium plus 20 g/L of glycerol) and incubated for 120 h under the same conditions. After removing the bacteria by centrifugation, the fermentation broth was mixed well with an equal volume of ethyl acetate, and centrifuged to get the supernatant which was then evaporated to dryness under nitrogen. The residues were dissolved in 50 % methanol-water solution, filtered through a 0.22 μm filter for analysis.

The OD_600_ values of cell cultures of each experiment were measured using microplate reader (Bio Tek, Synergy H1, USA) with an interval of 24 h, as shown in Figs. S2, S3, S4, and S5. All experiments were carried out in technical triplicate and at least biological duplicate. The yield is presented as mean ± SD. The production stability of the corresponding strain was verified through repeating the fermentation at least three times.

### 2.6 HPLC conditions

An Agilent 1260 HPLC equipped with a Phenomenex Luna C18 column (4.6 mm × 250 mm, 5 μm) and a UV detector were used to perform HPLC analysis. The HPLC conditions for determining L-Phe were as follows: Solvent A = water; Solvent B=CH_3_CN; Column temperature = 40 °C; Flow rate = 0.5 mL/min: 0–6 min, 90 % A and 10 % B to 90 % A and 10 % B; 6–18 min, 90 % A and 10 % B to 40 % A and 60 % B. L-Phe was detected at 210 nm.

The HPLC conditions for determining L-Tyr were as follows: Solvent A = 8 mmol/L of KH_2_PO_4_ in water; Solvent B=CH_3_OH; Column temperature = 35 °C; Flow rate = 1 mL/min: 0–8 min, 90 % A and 10 % B to 90 % A and 10 % B. L-Tyr was detected at 280 nm.

The HPLC conditions for determining PA, CA, 1-OPCG, and 1-*O*-caffeoylglycerol (1-OCG) were set as follows: column temperature at 35 °C and flow rate at 1 mL/min. A gradient mobile phase was applied using Solvent A (0.1 % formic acid in water) and Solvent B (acetonitrile) as follows: 0–10 min, 80 % A/20 % B to 60 % A/40 % B; 10–15 min, 60 % A/40 % B to 20 % A/80 % B; 15–20 min, 20 % A/80 % B to 80 % A/20 % B. PA, CA, 1-OPCG, and 1-OCG were detected at 310 nm, 324 nm, 312 nm, and 312 nm, respectively.

### 2.7 UPLC-QTOF-MS conditions

UPLC-QTOF-MS analysis was performed using an AB SCIEX Triple TOF® 5600+ High Resolution Mass Spectrometer (AB Sciex, USA), LC-30AD High Performance Liquid Chromatograph (Shimadzu, Japan) for the determination of 1-OPCG and 1-OCG. Analytes were separated using ACQUITY Premier HSS T3 1.8 μm VanGuard FIT (2.1 mm × 100 mm). The chromatographic conditions were: Solvent A = 0.1 % HCO_2_H in water; Solvent B=CH_3_OH; Column temperature = 35 °C; Flow rate = 0.2 mL/min; Injection volume = 2 μL: 0–2 min, 100 % A and 0 % B to 90 % A and 10 % B; 2–10 min, 90 % A and 10 % B to 60 % A and 40 % B; 10–20 min, 60 % A and 40 % B to 30 % A and 70 % B; 20–22 min, 30 % A and 70 % B to 30 % A and 70 % B. The mass spectrometry conditions were as follows: electrospray ion source (ESI) source in negative ion mode; ion spray voltage set to −4.5 kV; scanning range from *m/z* 100 to 1500; ion source temperature at 500 °C; ion source gas 1 and gas 2 both set to 50 psi; curtain gas at 35 psi; collision energy at −5 V; and declustering potential at −80 V.

### 2.8 UPLC-MS/MS conditions

UPLC-MS/MS analysis was performed using an AB SCIEX QTRAP® 6500 mass spectrometer (AB Sciex, USA), LC-30AD high performance liquid chromatograph (Shimadzu, Japan) to quantify the target product. Analytes were separated using a ACQUITY Premier HSS T3 1.8 μm VanGuard FIT (2.1 mm × 100 mm). The chromatographic conditions were: Solvent A = 0.1 % HCO_2_H in water; Solvent B=CH3OH; Column temperature = 40 °C; Flow rate = 0.2 mL/min; Injection volume = 3 μL: 0–2 min, 100 % A and 0 % B to 90 % A and 10 % B; 2–10 min, 90 % A and 10 % B to 60 % A and 40 % B; 10–20 min, 60 % A and 40 % B to 30 % A and 70 % B; 20–25 min, 30 % A and 70 % B to 100 % A and 0 % B. The mass spectrometry conditions were: electrospray ion source (ESI), negative ion mode scanning, ionspray voltage floating was −4.5 kV, ion source temperature was 500 °C, ion source gas 1 and ion source gas 2 were both 35 psi, curtain gas was 35 psi, collision energy was −26.46 V, and declustering potential was −33.96 V. 1-OPCG has a Q1 mass of 237.2 Da, a Q3 mass of 144.9 Da.

### 2.9 Adenosine triphosphate assay

Enhanced ATP assay kit (S0027, Beyotime, China) was used to detect the intracellular ATP levels. Enhanced BCA Protein Assay Kit (P0010, Beyotime, China) was used to detect the protein concentration in samples.

### 2.10 Subculture experiments for stability evaluation of engineered *S. cerevisiae* strains

Subculture experiments were performed to evaluate the stability of HYC13 and HYC20. To do that, the obtained positive recombinant strains were cultured in YPD liquid medium, and fresh YPD medium was replaced every 24 h for 15 consecutive days. After the subculture experiment, PCR was used to detect the gene expression cassette. The sequencing results also confirmed consistency before and after.

## 3 Results and discussion

### 3.1 Engineering the upstream metabolic flux to promote L-Tyr and L-Phe supply

The precursors for HCGs biosynthesis are derived from both L-Tyr-pathway and L-Phe-pathway (Fig. 1). Meanwhile, enhancing acetyl-coenzyme A (acetyl-CoA) will facilitate the generation of ATP (Fig. 1). In order to improve the supply of L-Tyr and L-Phe precursors, as well as acetyl-CoA and ATP, *S. cerevisiae* chassis cell was engineered to facilitate the upstream metabolic flux to these molecules by co-expressing heterologous *LmXfpk* and *CkPta* combined with *ScARO4*^*K229L*^, *ScARO7*^*G141S*^, and *EcAROL* genes. *LmXfpk* (encoding phosphoketolase, XFPK) and *CkPta* (encoding phosphotransacetylase, PTA) are responsible for the formation of erythrose-4-phosphate (E4P) and acetyl-CoA, two important metabolic intermediates that plays a central role in energy metabolism and various biochemical reactions [11,17] (Fig. 1). *ScARO4*^*K229L*^ and *ScARO7*^*G141S*^ are the feedback-inhibition-resistant alleles of *ARO4* and *ARO7*, respectively. Expression of the *E. coli*-derived shikimate kinase gene *EcAROL* to increase the metabolic flux of the shikimic acid pathway [14] (Fig. 1).

**Fig. 2:**
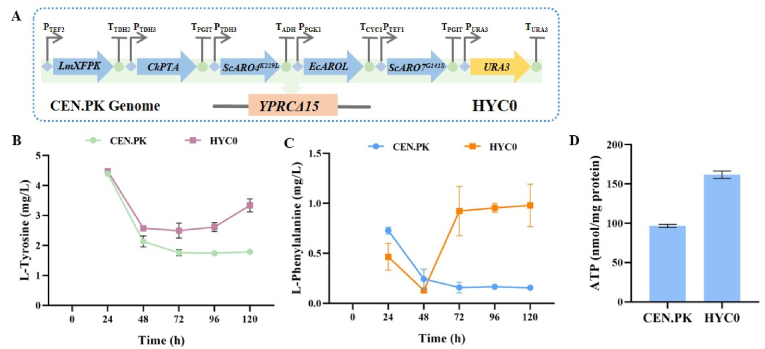
**Scheme of genetic modification in *S. cerevisiae* strain HYC0 and the concentration of L-Tyr and L-Phe in the strain. (A) Scheme of HYC0. (B) l-Tyrosine concentration. (C) l-Phenylalanine concentration. (D) ATP level.**

To do that, the expression cassettes separately containing heterologous *LmXfpk*, *CkPta*, *ScARO4*^*K229L*^, *ScARO7*^*G141S*^, and *EcAROL* genes combined with the selection marker *URA3* were integrated into the YPRC△15 site of Chromosome XVI of *S. cerevisiae* CEN.PK2–1C genome considering this site exhibited the highest transcriptional activity among 20 different integration sites [33], resulting in the *S. cerevisiae* strain HYC0 (Fig. 2A). As shown in Fig. 2, the intracellular concentration of L-Tyr and L-Phe in the engineered yeast strain HYC0 was 0.98 ± 0.21 mg/L and 3.34 ± 0.22 mg/L, 6.24-fold and 1.96-fold of those in *S. cerevisiae* CEN.PK strain, respectively. This result proved that co-expression of heterologous *LmXfpk* and *CkPta* combined with *ScARO4*^*K229L*^, *ScARO7*^*G141S*^, and *EcAROL* significantly enhanced the capability of *S. cerevisiae* to synthesize L-Tyr and L-Phe. It was reported that wild-type *S. cerevisiae* could produce L-Tyr [34], and expressing *Bbxfpk*, *Ckpta*, *Aro4*^*K229L*^, and *Aro7*^*G141S*^ enhanced the production of PA by 18 % [11]. The improved intracellular accumulation of L-Tyr and L-Phe in HYC0 in this study provides a more favorable conditions for the production of PA and 1-OPCG in *S. cerevisiae* strain HYC0.

It should be mentioned that L-Tyr levels in the strain decreased after 24 h. This trend was similar to the reported in the literature [35]. It was possible that the intracellular L-Tyr had been transported outside the cell via the L-Tyr transport system or had been used to synthesize other bioactive substances. The contents of L-Tyr and L-Phe in HYC0 continued to increase after 72 h, and reached the highest at 120 h. Therefore, in subsequent experiments, we set the fermentation time of the strains to 120 h.

In addition, the intracellular ATP level of HYC0 cells was 161.76 ± 4.73 nmol/mg protein, 1.67-fold of that in CEN.PK (Fig. 2D). This result demonstrated that co-expression of heterologous *LmXfpk* and *CkPta* significantly enhanced ATP supply in *S. cerevisiae*, indicating that acetyl CoA indirectly supported ATP generation through the TCA cycle.

### 3.2 Engineering the biosynthesis of PA in *S. cerevisiae*

**Fig. 3:**
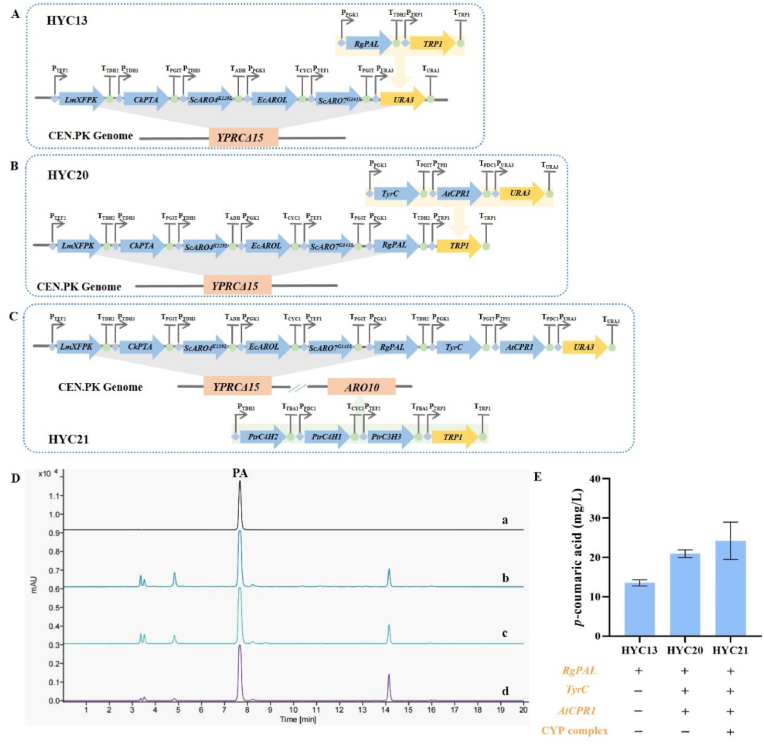
**Scheme of the genetic modifications in *S. cerevisiae* and d*e novo* biosynthesis of PA. (A) Scheme of HYC13. (B) Scheme of HYC20. (C) Scheme of HYC21. (D) Typical HPLC profile of products in yeast strain HYC13, HYC 20, and HYC 21. Line a: PA standard. Line b: Products in HYC13. Line c: Products in HYC20. Line d: Products in HYC21. (E) Production of PA in HYC13, HYC 20, and HYC 21.**

In order to recreate the PA synthetic pathway in *S. cerevisiae* strain, the *Rhodotorula glutinis* phenylalanine ammonia lyase gene (*RgPAL*), the *A. thaliana* cytochrome P450 reductase gene (*AtCPR1*), and the feedback-insensitive *Zymomonas mobilis* cyclohexadienyl dehydrogenase gene (*ZmTyrC*) [36,37], were used. *RgPAL* was integrated into HYC0 genome to generate strain HYC13, using the URA3 selection marker as the targeted integration site (Fig. 3A). Next, *AtCPR1* and *ZmTyrC* were integrated into the genome of strain HYC13 to generate strain HYC20, using the *TRP1* selection marker as the target integration site (Fig. 3B). Subsequently, a CYP complex, comprising the expression cassettes of *PtrC4H1*, *PtrC4H2*, and *PtrC3H3*, was integrated into the *ARO10* locus on chromosome IV of the HYC20 genome, generating the yeast strain HYC21 (Fig. 3C). This genetic modification combined the L-Tyr derived pathway and the L-Phe derived pathway to produce PA and CA, while simultaneously knocking out the *ARO10* gene to reduce the fusel alcohol/acid byproducts (Fig. 1).

These engineered yeast strains were used to do fermentation in shake flasks. As shown in Fig. 3D and E, all the three strains produced PA, and the concentration of PA in HYC13 was 13.53 ± 0.80 mg/L, that in HYC20 and HYC21 was 20.91 ± 0.96 mg/L and 24.21 ± 4.72 mg/L, 1.55- and 1.79-fold of HYC13, respectively. Meanwhile, CA in HYC21 was also examined but was undetectable (Fig. S6). These results indicated that the recreated PA/CA synthetic pathway has the capability to *de novo* biosynthesis of PA but not CA, and heterologous expression of *ZmTyrC*, or co-expression of *AtCPR1* and CYP complex facilitated the biosynthesis of PA. It was noted that the difference in PA between HYC21 and HYC20 strains was not significant, which may be due to the knockout of *ARO10* or the introduction of CYP complex, disrupting the original metabolic flux balance and introducing a certain metabolic burden. The absence of CA in HYC21 may be due to the inactivity of PtrC3H3 in this system. Therefore, this article focused on the *de novo* biosynthesis of 1-OPCG.

### 3.3 Biosynthesis of HCGs using *S. cerevisiae* modified with *OsHCT4* and 4CL

**Fig. 4:**
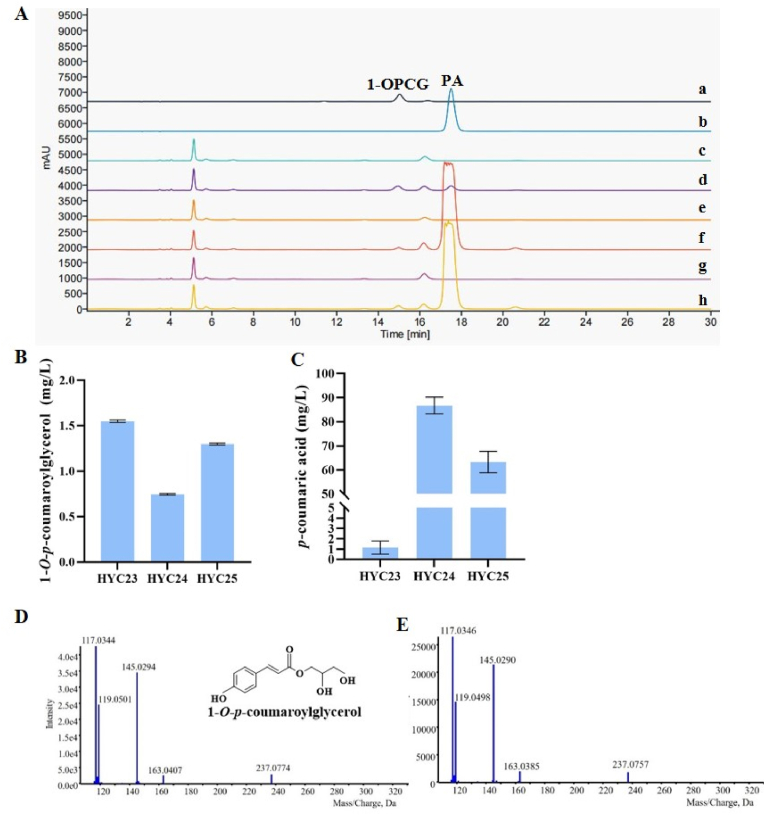
**Fermentation results of HYC23-25 by addition of PA and glycerol.** (A) Typical HPLC profiles of the products of HYC23, HYC24, and HYC25. Line a: 1-OPCG standard. Line b: PA standard. Line c, e, g: HYC23, HYC24, and HYC25 without PA or glycerol. Line d, f, h: HYC23, HYC24, and HYC25 with addition of PA and glycerol. (B) Concentration of 1-OPCG in HYC23, HYC24, and HYC25. (C) Concentration of PA in HYC23, HYC24, and HYC25. (D) MS-MS chromatogram of 1-OPCG standard. (E) MS-MS chromatogram of 1-OPCG from strain HYC23.

In order to screen for the optimal 4CL-HCT module for the biosynthesis of HCGs, *OsHCT4* was selected and separately combined with *At4CL1*, *Pc4CL1*, and *Sm4CL2* (Table 3), to build three combinations, *OsHCT4*-*At4CL1*, *OsHCT4*-*Pc4CL1*, and *OsHCT4*-*Sm4CL2*, resulting in three engineered yeast strains HYC23, HYC24, and HYC25, respectively. Upon the addition of 100 mg/L PA and 25 g/L glycerol, all the three *S. cerevisiae* strains produced 1-OPCG (Fig. 4A). Among them, HYC23, containing *At4CL1* and *OsHCT4*, exhibited the highest yield, reaching 1.55 ± 0.01 mg/L (Fig. 4B). PA content at the fermentation endpoint was also measured. HYC23 produced 1.14 ± 0.64 mg/L, significantly lower than HYC24 and HYC25, which yielded 86.73 ± 3.51 mg/L and 63.27 ± 4.49 mg/L of PA, respectively (Fig. 4C), suggesting that HYC23 had a higher capacity to convert PA into 1-OPCG than HYC24 and HYC25. Since 1-OPCG has distinct HPLC behavior compared to 2-*O*-*p*-coumaroyl glyceride (2-OPCG) [25] and the product of HYC23 showed the same fragmentation pattern as the 1-OPCG standard in MS/MS analysis (Fig. 4D and E), the product was confirmed as 1-OPCG.

**Fig. 5:**
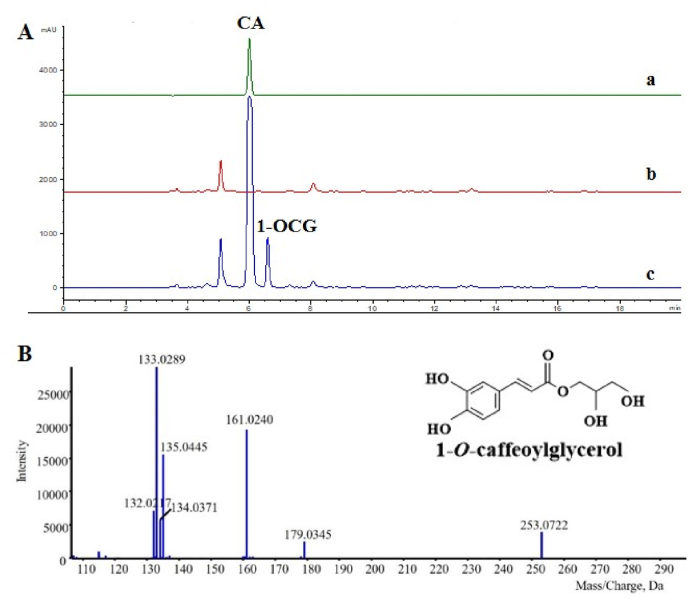
**Fermentation results of HYC23 by addition of CA and glycerol.** (A) Typical HPLC profiles of the products from yeast strain HYC23. Line a: CA standard. Line b: HYC23 without CA or glycerol. Line c: HYC23 with addition of CA or glycerol. (B) MS-MS chromatogram of 1-OCG from strain HYC23.

Meanwhile, when 100 mg/L CA and 25 g/L glycerol were supplemented to the HYC23 strain, a new product was produced (Fig. 5A). MS/MS analysis revealed that the product in HYC23 had a *m/z* of 254, with a fragment ion at *m/z* 180, consistent with the molecular mass of CA. Similarly, the molecular weight of the reaction product was increased by 74 Da relative to that of CA, indicating that OsHCT4 catalyzes an acyl transfer reaction from CA-CoA to glycerol (Fig. 5B). MS/MS analysis confirmed it is 1-OCG by comparing the *m/z* and the fragmentation pattern with literature [5]. Since the 1-OCG reference standard is unavailable, it was not quantified.

These results demonstrated that co-expression of *OsHCT4* and 4CL in *S. cerevisiae* enabled the biosynthesis of HCGs. Among them, the *At4CL1* and *OsHCT4* was the best combination to catalyze the conversion of PA to 1-OPCG. Then, *At4CL1* and *OsHCT4* were used for the next targeted integration into *S. cerevisiae* genome.

### 3.4 *De novo* biosynthesis of HCGs in engineered *S. cerevisiae*

**Fig. 6:**
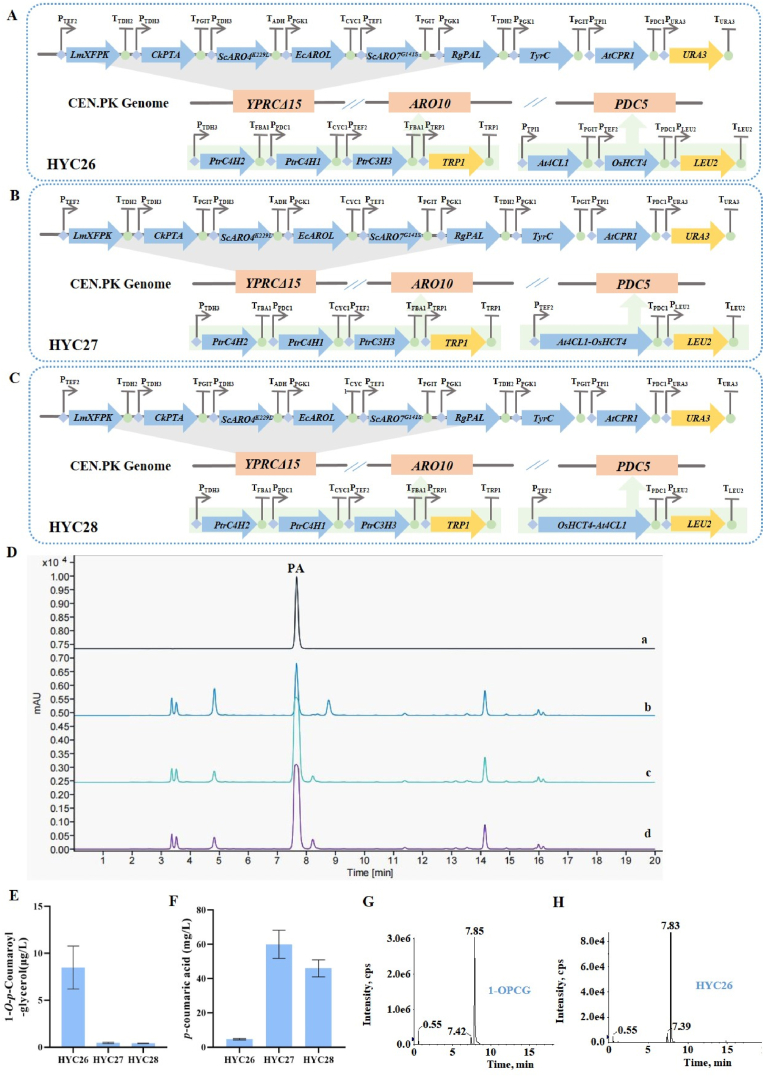
**Scheme of the genetic modifications in *S. cerevisiae* and d*e novo* biosynthesis of 1-OPCG. (A) Scheme of HYC26. (B) Scheme of HYC27. (C) Scheme of HYC28. (D) Typical HPLC profiles of the products of HYC26, HYC27, and HYC28. Line a: PA standard. Line b–d: HYC26-28. (E) Production of 1-OPCG in HYC26, HYC 27, and HYC 28. (F) Production of PA in HYC26, HYC 27, and HYC 28. (G) UPLC-MS/MS analysis chromatogram of 1-OPCG standard. (H) UPLC-MS/MS analysis chromatogram of 1-OPCG from strain HYC26.**

To enable *de novo* biosynthesis of 1-OPCG in *S. cerevisiae*, the expression cassettes of *At4CL1* and *OsHCT4*, as well as two fusion proteins, *At4CL1-OsHCT4* and *OsHCT4-At4CL1*, were employed to construct the 4CL-HCT module. These constructs were individually integrated into the *PDC5* locus of strain HYC21, generating strains HYC26, HYC27, and HYC28 (Fig. 6A, B, C), aiming to simultaneously reduce the production of by-products, aromatic alcohols, and direct metabolic flux to aromatic amino acids, as supported by the literature [12].

After fermentation in shake flasks for 120 h, the concentration of 1-OPCG in HYC26 was 8.49 ± 2.29 μg/L, and that in HYC27 and HYC28 was only 0.46 ± 0.07 μg/L and 0.41 ± 0.02 μg/L (Fig. 6E). By comparing the retention times in the UPLC-MS/MS profiles, it demonstrated the product of HYC26 was 1-OPCG (Fig. 6G and H). These results indicated that the integration of the 4CL-HCT module into PA-producing strains led to *de novo* biosynthesis of HCGs, combining with engineering the upstream metabolic flux. Among them, the recreated 4CL-HCT module using individual *At4CL1* and *OsHCT4* expression cassettes produced a higher production of 1-OPCG, while the two fusion proteins resulted in lower yields. It is probably that spatial structures of the At4CL1 and OsHCT4 enzymes may undergo changes after fusion, leading to the occlusion or distortion of the original active sites. This, in turn, reduced the binding capacity of enzymes with its substrates, ultimately weakening or even abolishing the original function of the protein, as reported [38].

A significant difference in the PA conversion rate was observed between HYC26-28 and HYC23, which might be due to a relatively low copy number at the *PDC5* locus. Meanwhile, it was possible that the PA-CoA accumulated in HYC26-28 was not converted to 1-OPCG. We attempted to quantify PA-CoA using HPLC with a reversed-phase C18 column; however, no signal was detected, even for the PA-CoA standard (Fig. S7), likely due to its high polarity and hydrophilicity [39,40]. Given that PA-CoA, a common intermediate in phenylpropanoid metabolism, has typically not been measured in synthetic biology studies such as those on naringenin and rosmarinic acid biosynthesis [14,41], we did not pursue further analysis.

Interestingly, the concentration of PA in strains HYC27 and HYC28 reached 59.97 ± 8.17 mg/L and 46.00 ± 4.87 mg/L, 12.76 and 9.78 times HYC26, respectively (Fig. 6D–F). It was even higher than that in HYC21, which was 2.48 and 1.90 times of HYC21, respectively. It was speculated that this could be attributed to a reduction in the activity of the two fusion proteins, At4CL1-OsHCT4 and OsHCT4-At4CL1, thereby preventing the accumulated PA from being converted to 1-OPCG.

### 3.5 Stability evaluation of the engineered *S. cerevisiae* strains

As shown in Fig. S8, the band sizes of the amplification products in the agarose gel electrophoresis images before and after subculturing are correct, and the sequencing results also confirm the consistency before and after the consecutive subculturing. This indicates that both HYC13 and HYC20 have good genetic stability.

## 4 Conclusions

In this study, we constructed a *de novo* biosynthetic pathway of HCGs by optimizing a PA-producing module and a 4CL-HCT module. Meanwhile, the upstream metabolic flux was engineered to improve the L-Tyr and L-Phe precursors, as well as acetyl-CoA and ATP supply by targeted integration of special expression cassettes into the YPRCΔ15, *ARO10*, and *PDC5* loci of *S. cerevisiae* chromosome. Eventually, the engineered strain produced 8.49 ± 2.29 μg/L of 1-OPCG in shake flask fermentation.

Compared with the production of 1-OPCG via the biotransformation of PA using *E. coli* co-expressing *Os4CL* and *OsHCT4* by the addition of PA as a substrate [25], this study provides a more practical foundation for 1-OPCG production via a *de novo* biosynthesis platform. In future, several strategies will be employed to enhance the 1-OPCG production for industrial applications. Regarding biosynthesis, the synthesis pathway of 1-OPCG will be optimized by employing strategies such as the *GAL*p expression system [11,13,14], cofactor engineering [17,42,43], screening and engineering HCT [44,45]. Additionally, a more favorable fermentation process for 1-OPCG synthesis will be developed through the optimization of medium ingredients and fermentation conditions [46].

## CRediT authorship contribution statement

**Haiyan Zou:** Writing – review & editing, Writing – original draft, Visualization, Methodology, Investigation, Formal analysis, Data curation, Conceptualization. **Chuanguang Xiao:** Resources, Methodology. **Shujuan Zhao:** Writing – review & editing, Supervision, Project administration, Funding acquisition, Conceptualization.

## Availability of data and materials

All data generated or analyzed during this study are included in this research article.

## Declaration of competing interest

The authors declare that they have no known competing financial interests or personal relationships that could have appeared to influence the work reported in this paper.
