# Supplementary material for: Metabolic engineering Saccharomyces cerevisiae for de novo biosynthesis of hydroxycinnamoyl glycerols
Source: Synth Syst Biotechnol. 2025 Jul 17;10(4):1257–66. doi: 10.1016/j.synbio.2025.07.005 (PMC12318264; doi:10.1016/j.synbio.2025.07.005)
Supplement: Multimedia component 1 [file mmc1.pdf]

Supplementary Materials

Metabolic engineering *Saccharomyces cerevisiae* for *de novo* biosynthesis of hydroxycinnamoyl glycerols

Haiyan Zou, Chuanguang Xiao, Shujuan Zhao*

The SATCM Key Laboratory for New Resources & Quality Evaluation of Chinese Medicine, The MOE Key Laboratory for Standardization of Chinese Medicines and Shanghai Key Laboratory of Compound Chinese Medicines, Institute of Chinese Materia Medica, Shanghai University of Traditional Chinese Medicine, Shanghai 201203, China

*Corresponding author. E-mail address: zhaoshujuan@126.com, zhaoshujuan@shutcm.edu.cn.

ORCID: 0000-0002-2269-925X

Table S1 Primers used in this study

Primers	Sequences（5'→3' ）
Primers for vector construction	
P_{TEF2}-*Kpn* I-F	GGTACCCGCCATAACCAAGGTATCTA
P_{TEF2}-*Xho* I-R	CTCGAGAATGACCGGTAGTGGTAGAC
T_{TDH2}-*BamH* I-F	GGATCCGGCTTAAATTTAACTCCTTA
T_{TDH2}-*Not* I-R	GCGGCCGCGCGAAAAGCCAATTAGTGTG
P_{TDH3}-*Kpn* I-F	GGTACCatactagcgttgaatgttag
P_{TDH3}-*Xho* I-R	CTCGAGttgtttgtttatgtgtgttt
T_{PGIT}-*BamH* I-F	GGATCCaacaaatcgctcttaaatat
T_{PGIT}-*Not* I-R	GCGGCCGCGGTATACTGGAGGCTTCATG
P_{TDH3}-*Kpn* I-F	GGTACCatactagcgttgaatgttag
P_{TDH3}-*Xho* I-R	CTCGAGttgtttgtttatgtgtgtttattc
ARO4^{K229L}-*Xho* I-F	CTCGAGATGAGTGAATCTCCAATGTT
ARO4K^{229L}-*BamH* I-R	GGATCCCTATTTCTTGTTAACTTCTC
T_{ADH}-*BamH* I-F	GGATCCaactaagcgaatttcttatga
T_{ADH}-*Not* I-R	GCGGCCGCCCGTGTGGAAGAACGATTAC
P_{TEF1}-*Kpn* I-F	GGTACCgtacCAGTGATCCCCCACAC
P_{TEF1}-*Xho* I-R	CTCGAGtcgaGtttgtaattaaaact
ARO7^{G141S}-*Xho* I-F	CTCGAGATGGATTTCACAAAACCAGA
ARO7^{G141S}-*BamH* I-R	GGATCCTTACTCTTCCAACCTTCTTA
T_{PGIT}-*BamH* I-F	GGATCCaacaaatcgctcttaaatat
T_{PGIT}-*Not* I-R	GCGGCCGCGGTATACTGGAGGCTTCATG
P_{PGK1}-*Kpn* I-F	GGTACCACGCACAGATATTATAACAT
P_{PGK1}-*Xho* I-R	CTCGAGtgttttatatttgttgtaaa

AROL-Xho I-F	CTCGAGATGACACAACCTCTTTTTCT
AROL-BamH I-R	GGATCCTCAACAATTGATCGTCTGTG
T_{CYC1}-*BamH* I-F	GGATCCTcatgtaattagttatgtca
T_{CYC1}-*Not* I-R	GCGGCCGCgcaaattaaagccttcgagc
Primers for site directed mutagenesis of *ARO4* and *ARO7*	
ARO4-F	ATGAGTGAATCTCCAATGTT
ARO4-R	CTATTTCTTGTTAACTTCTCTTCT
ARO4^{K229L}-F	CATTTCATGGGTGTTACTTTGCATGGTGTTGCTGCTATC
ARO4^{K229L}-R	GATAGCAGCAACACCATGCAAAGTAACACCCATGAAATG
ARO7-F	ATGGATTTCACAAAACCAGAAACTG
ARO7-R	TTACTCTTCCAACCTTCTTAGCAA
ARO7^{G141S}-F	GATAAGAATAACTTCAGTTCTGTTGCCACTAG
ARO7^{G141S}-R	CTAGTGGCAACAGAACTGAAGTTATTCTTATC
Primers for construction of the PA-producing module	
For amplification of promoters and terminators	
P_{PGK1}-F	ctatagggcgaattgggtaccACGCACAGATATTAT
P_{PGK1}-R	atcgataccgtcgacctcgagtgttttatatttgtt
T_{TDH2}-F	ttcctgcagcccgggggatccGGCTTAAATTTAACT
T_{TDH2}-R	agctccaccgcggtggcggccgcGCGAAAAGCCAATTA
Primers for construction of the 4CL-HCT module	
For amplification of *4CL* expression cassettes	
At4CL1-F	CATTAAGGAAATTGTAagcttATGGCGCCACAAGAA
At4CL1-R	TAAGAGCGATTTGTTGGATCcTCACAATCCATTTGC
Pc4CL1-F	CATTAAGGAAATTGTAagcttATGGGAGATTGTGTA
Pc4CL1-R	TAAGAGCGATTTGTTGGATCcTTATTTGGGAAGATC

Sm4CL2-F	CATTAAGGAAATTGTAagcttATGGAGGTTCCCACG
Sm4CL2-R	TAAGAGCGATTTGTTGGATCcttagactgcagctgc
P_{TPI1}-*Xho* I-F	cTCGAGTATAATAATCCTACGTTAGTGTGA
P_{TPI1}-*Hind III*-R	aagctTACAATTTCCTTAATGGATTGTTTG
T_{PGIT}-*BamH* I-F	GGATCCaacaaatcgctcttaaatat
T_{PGIT}-*Not* I-R	GCGGCCGCGGTATACTGGAGGCTTCATG
For amplification of *OsHCT4* expression cassettes	
T_{PDC1}-*BamH* I-F	GGATCCgcgatttaatctctaatta
T_{PDC1}-*Not I*-F	gcggccGCtgttccttaatcaagga
P_{TEF2}-*Kpn* I-F	GGTACCCGCCATAACCAAGGTATCTA
P_{TEF2}-*Xho* I-R	CTCGAGAATGACCGGTAGTGGTAGAC
Primers for expression cassettes construction	
For construction of the PA-producing module	
For amplification of *TyrC* expression cassettes	
P_{PGK1}-F	ACGCACAGATATTATAACATCTGCACaataggcatttgcaagaattactcgtgagtaag
TyrC-P_{PGK1}-R	ACCCAAACCAATAATAGCAATATGCTTGAAAACAGTCATtgttttatatttgttgtaaa
P_{PGK1}-*TyrC*-F	aggaagtaattatctactttttacaacaaatataaaacaATGACTGTTTTCAAGCATAT
T_{PGIT}-*TyrC*-R	TTTAATGTTCTTTAGGTATATATTTAAGAGCGATTTGTTTTATGGATGAATATCATGAT
TyrC-T_{PGIT}-F	AGATTAGCTTTGAAAACTGATCATGATATTCATCCATAAAACAAATCGCTCTTAAATAT
T_{PGIT}-R	GGTATACTGGAGGCTTCATGAGTTATGTCCTTCGCGCACTGATTCATCTTTGAAACTAA
For amplification of AtCPR1 expression cassettes	
P_{TPI1}-F	TATAATAATCCTACGTTAGTGTGAGCGGGATTTAAACTGTGAGGACCTTAATACATTCA
AtCPR1-P_{TPI1}-R	CTGTTTAAACAAATCAGATGCATATAAAGCAGAAGTCATACAATTTCCTTAATGGATTG
P_{TPI1}-*AtCPR1*-F	TAAATTAAACGGTTCCAAACAATCCATTAAGGAAATTGTATGACTTCTGCTTTATATGC
T_{PDC1}-*AtCPR1*-R	gcttataaaactttaactaataattagagattaaatcgcTTACCAAACATCTCTCAAAT
AtCPR1-T_{PDC1}-F	TTACAAACTGAAGGTAGATATTTGAGAGATGTTTGGTAAgcgatttaatctctaattat

T_{PDC1}-R	tgttccttaatcaaggatacctctttttttttccttggttccactaattcatcggtttt
For amplification of CYP complex expression cassettes	
ARO10up-P_{TDH3}-F	GTCGATAATGTTATCCGCGATATTTACAAGTATTCTAAAatactagcgttgaatgttag
P_{TDH3}-R	GGAACCTAATAATGTTTTTTCTAACAACAACAAATCCATttgtttgtttatgtgtgttt
PtrC4H2-F	caagaacttagtttcgaataaacacacataaacaaacaaATGGATTTGTTGTTGTTAGA
PtrC4H2-R	cattaaaaaactatatcaattaatttgaattaacGGATCTTAAAATGATCTTGGTTTAG
T_{FBA1}-F	AAACATTCAACTATAGTTGCTAAACCAAGATCATTTTAAGATCCgttaattcaaattaa
P_{PDC1}-T_{FBA1}-R	ACATCACATCAGCGGAACATATGCTCACCCAGTCGCATGctagTgctatcaaaaacgat
T_{FBA1}-P_{PDC1}-F	aagtcatcctaatcgatctatcgtttttgatagcActagCATGCGACTGGGTGAGCATA
P_{PDC1}-R	AGAACCCAATAAAGTTTTTTCTAATAATAACAAATCCATTTTGATTGATTTGACTGTGT
PtrC4H1-F	CATAACCTCACGCAAAATAACACAGTCAAATCAATCAAAATGGATTTGTTATTATTAGA
PtrC4H1-R	GAGGGCGTGAATGTAAGCGTGACATAACTAATTACATGATTAAAAAGATCTTGGTTTAG
T_{CYC1}-F	AAACATTCAACTATAGTAGCTAAACCAAGATCTTTTTAATCATGTAATTAGTTATGTCA
P_{TEF2}-T_{CYC1}-R	TTTGCTGATTGGCGGTCTATAGATACCTTGGTTATGGCGGCAAATTAAAGCCTTCGAGC
T_{CYC1}-P_{TEF2}-F	CTTGAGAAGGTTTTGGGACGCTCGAAGGCTTTAATTTGCCGCCATAACCAAGGTATCTA
P_{TEF2}-R	TAAAGTAATAAAAGAAATTGGAATTAATAATAAGTTCATAATGACCGGTAGTGGTAGAC
PtrC3H3-F	GTCATGTCGATTCTGGTAAGTCTACCACTACCGGTCATTATGAACTTATTATTAATTCC
PtrC3H3-R	tcattaaaaaactatatcaattaatttgaattaacGGATCTTAAATATCAACTGCAACT
T_{FBA1}-F	CCATCACATTTGTATAAAAGAGTTGCAGTTGATATTTAAGATCCgttaattcaaattaa
P_{TRP1}-T_{FBA1}-R	attaaatgcttcctatattatatatatagtaatgtcgttctagTgctatcaaaaacgat
Primers for targeted integration of expression cassettes through homologous recombination	
Enhancing the supply of L-Tyrosine and L-Phenylalanine precursor	
ZH-20up-F	GCCAGGCGCCTTTATATCATataattaagacacaa
ZH-20up-R	TTTGCTGATTGGCGGTCTATAGATACCTTGGTTATGGCGtttgcgaaaccctatgctct
ZH-*LmXFPK*-F	aatttcaaatccgaacaacagagcatagggtttcgcaaaCGCCATAACCAAGGTATCTA
ZH-*LmXFPK*-R	aaacttcttgttgttgacgctaacattcaacgctagtatGCGAAAAGCCAATTAGTGTG

ZH-*CkPTA*-F	TCGATAAAGCACTTAGTATCACACTAATTGGCTTTTCGCatactagcgttgaatgttag
ZH-*CkPTA*-R	aaacttcttgttgttgacgctaacattcaacgctagtatGGTATACTGGAGGCTTCATG
ZH-*ARO4*^{K229L}-F	gtgcgcgaaggaCATAACTCATGAAGCCTCCAGTATACCatactagcgttgaatgttag
ZH-*ARO4*^{K229L}-R	gcaaatgcctattGTGCAGATGTTATAATATCTGTGCGTCCGTGTGGAAGAACGATTAC
ZH-*AROL*-F	tcagaggacaacacctgTTGTAATCGTTCTTCCACACGGACGCACAGATATTATAACAT
ZH-*AROL*-R	aacattttgaagcTATGGTGTGTGGGGGATCACTGgtacgcaaattaaagccttcgagc
ZH-*ARO7*^{G141S}-F	cttgagaaggttttgggacgctcgaaggctttaatttgcgtacCAGTGATCCCCCACAC
ZH-*ARO7*^{G141S}-R	atacatTATACGAAGTTATtcgacgatctagtcagtctAGGTATACTGGAGGCTTCATG
ZH-URA3-F	gtgcgcgaaggaCATAACTCATGAAGCCTCCAGTATACCTagactgactagatcgtcga
ZH-URA3-R	cttcttagtgcttgtatatgctcatcccgaccttccattAGagtctgcttagctatgat
ZH-20down-F	tatgcTATACGAAGTTATtatcatagctaagcagactCTaatggaaggtcgggatgagc
ZH-20down-R	ATAAAGCAGCCGCTACCAAAcagacaagattcagt
Primers for targeted integration of the PA-producing module into the genome of *S. cerevisiae*	
For targeted integration of the *RgPAL/Trp1* cassettes	
ARO7-F	TGTTGCTGAAGCCAAGTTCCAATCGGATATCCCGCTATACACAAAGCTGATCAAAAGTA
P_{PGK1}-T_{PGIT}-R	gcaaatgcctattGTGCAGATGTTATAATATCTGTGCGTGGTATACTGGAGGCTTCATG
T_{PGIT}-P_{PGK1}-F	gtgcgcgaaggaCATAACTCATGAAGCCTCCAGTATACCACGCACAGATATTATAACAT
TRP1-T_{TDH2}-R	attaaatgcttcctatattatatatatagtaatgtcgttGCGAAAAGCCAATTAGTGTG
T_{TDH2}-TRP1-F	TCGATAAAGCACTTAGTATCACACTAATTGGCTTTTCGCaacgacattactatatatat
20down-TRP1-R	cttcttagtgcttgtatatgctcatcccgaccttccattcgaatggcgcgacgcgccct
TRP1-20down-F	gcgcttaatgcgccgctacagggcgcgtcgcgccattcgaatggaaggtcgggatgagc
20down-R	ATAAAGCAGCCGCTACCAAAcagacaagattcagtatgtaaggtaaatacctttttgca
For targeted integration of the TyrC/AtCPR1/URA3 cassettes	
RgPAL-F	GAAGGTTGCTTGTGCTGAAAAAGCTATTGCTTTGACTAGAACTGTTAGAGATTCTTTCT
P_{PGK1}-T_{TDH2}-R	gcaaatgcctattGTGCAGATGTTATAATATCTGTGCGTGCGAAAAGCCAATTAGTGTG

T_{TDH2}-P_{PGK1}-F	TCGATAAAGCACTTAGTATCACACTAATTGGCTTTTCGCACGCACAGATATTATAACAT
P_{TPI1}-T_{PGIT}-R	CAGTTTAAATCCCGCTCACACTAACGTAGGATTATTATAGGTATACTGGAGGCTTCATG
T_{PGIT}-P_{TPI1}-F	GTGCGCGAAGGACATAACTCATGAAGCCTCCAGTATACCTATAATAATCCTACGTTAGT
P_{URA3}-T_{PDC1}-R	aaatcaaaaaaaagaataaaaaaaaaatgatgaattgaatgttccttaatcaaggatac
T_{PDC1}-P_{URA3}-F	accaaggaaaaaaaaagaggtatccttgattaaggaacattcaattcatcattttttt
20down-R	ATAAAGCAGCCGCTACCAAAcagacaagattcagtatgtaaggtaaataccttttttgca
For targeted integration of the CYP complex/Trp1 cassettes	
ARO10-UP-F	ATGGCACCTGTTACAATTGAAAAGTTCGTAAATCAAGAAGAACGACACCTTGTTTCCAA
P_{TDH3}-*ARO10*up-R	aaacttcttgttgttgacgctaacattcaacgctagtatTTTAGAATACTTGTAAATAT
*ARO10*up-P_{TDH3}-F	GTCGATAATGTTATCCGCGATATTTACAAGTATTCTAAAatactagcgttgaatgttag
P_{PDC1}-T_{FBA1}-R	ACATCACATCAGCGGAACATATGCTCACCCAGTCGCATGctagTgctatcaaaaacgat
T_{FBA1}-P_{PDC1}-F	aagtcatcctaatcgatctatcgtttttgatagcActagCATGCGACTGGGTGAGCATA
P_{TEF2}-T_{CYC1}-R	TTTGCTGATTGGCGGTCTATAGATACCTTGGTTATGGCGGCAAATTAAAGCCTTCGAGC
T_{CYC1}-P_{TEF2}-F	CTTGAGAAGGTTTTGGGACGCTCGAAGGCTTTAATTTGCCGCCATAACCAAGGTATCTA
P_{TRP1}-T_{FBA1}-R	attaaatgcttcctatattatatatatagtaatgtcgttctagTgctatcaaaaacgat
T_{FBA1}-P_{TRP1}-F	aagtcatcctaatcgatctatcgtttttgatagcActagaacgacattactatatatat
*ARO10*down-T_{TRP1}-R	GAATTGAAAAGAGCCTGTTTCACAAACGACAACATCACCcgaatggcgcgacgcgccct
T_{TRP1}-*ARO10*down-F	gcgcttaatgcgccgctacagggcgcgtcgcgccattcgGGTGATGTTGTCGTTTGTGA
ARO10-DOWN-R	CTATTTTTTATTTCTTTTAAGTGCCGCTGCTTCAACCATGCACTTTAGCTGTTCGGGGA
Primers for targeted integration of the 4CL-HCT module into the genome of *S. cerevisiae*	
*PDC5*up-F	ATGTCTGAAATAACCTTAGGTAAATATTTATTTGAAAGATTGAGCCAAGTCAACTGTAA
P_{TPI1}-*PDC5*up-R	CAGTTTAAATCCCGCTCACACTAACGTAGGATTATTATAAACTTCAGCTTCAGCTTCAG
*PDC5*up-P_{TPI1}-F	TCTTTGAAGCCAAACGACGCTGAAGCTGAAGCTGAAGTTTATAATAATCCTACGTTAGT
P_{TEF2}-T_{PGIT}-R	TTTGCTGATTGGCGGTCTATAGATACCTTGGTTATGGCGGGTATACTGGAGGCTTCATG

T_{PGIT}-P_{TEF2}-F	GTGCGCGAAGGACATAACTCATGAAGCCTCCAGTATACCCGCCATAACCAAGGTATCTA
LEU2-T_{PDC1}-R	ataccgcacagatgcgtaaggagaaaataccgcatcaggtgttccttaatcaaggatac
T_{PDC1}-LEU2-F	accaaggaaaaaaaaagaggtatccttgattaaggaacacctgatgcggtattttctcc
*PDC5*down-LEU2-R	GAAGTTACCCAAATGGTTCCACATCCATTCTTGCTTCATtcgcgcgtttcggtgatgac
LEU2-*PDC5*down-F	tgtgtcagaggttttcaccgtcatcaccgaaacgcgcgaATGAAGCAAGAATGGATGTG
*PDC5*down-R	TTATTGTTTAGCGTTAGTAGCGGCAGTCAATTGAGCTTGTTTAACCAAGTTTTGTGGAG
P_{TEF2}-*PDC5*up-R	TTTGCTGATTGGCGGTCTATAGATACCTTGGTTATGGCGAACTTCAGCTTCAGCTTCAG
*PDC5*up-P_{TEF2}-F	TCTTTGAAGCCAAACGACGCTGAAGCTGAAGCTGAAGTTCGCCATAACCAAGGTATCTA
At4CL1-P_{TEF2}-R	CACCTGAGAAACTGCTTGTTCTTGTGGCGCCATCTCGAGAATGACCGGTAGTGGTAGAC
P_{TEF2}-*At4CL1*-F	GTCATGTCGATTCTGGTAAGTCTACCACTACCGGTCATTCTCGAGATGGCGCCACAAGA
linker-*At4CL1*-R	cgatccgccaccgccagagccacctccgcctgaaccgcctccaccCAATCCATTTGCTA
linker-*OsHCT4*-F	ggtggaggcggttcaggcggaggtggctctggcggtggcggatcgATGGCTACTGTTGA
linker-*OsHCT4*-R	cgatccgccaccgccagagccacctccgcctgaaccgcctccaccTGCAAGTCTAGCAA
linker-*At4CL1*-F	ggtggaggcggttcaggcggaggtggctctggcggtggcggatcgATGGCGCCACAAGA
T_{PDC1}-*At4CL1*-R	aaaactttaactaataattagagattaaatcgcGGATCCTCACAATCCATTTGCTAGTT
At4CL1-T_{PDC1}-F	GATCTGAGGGCAAAACTAGCAAATGGATTGTGAGGATCCgcgatttaatctctaattat

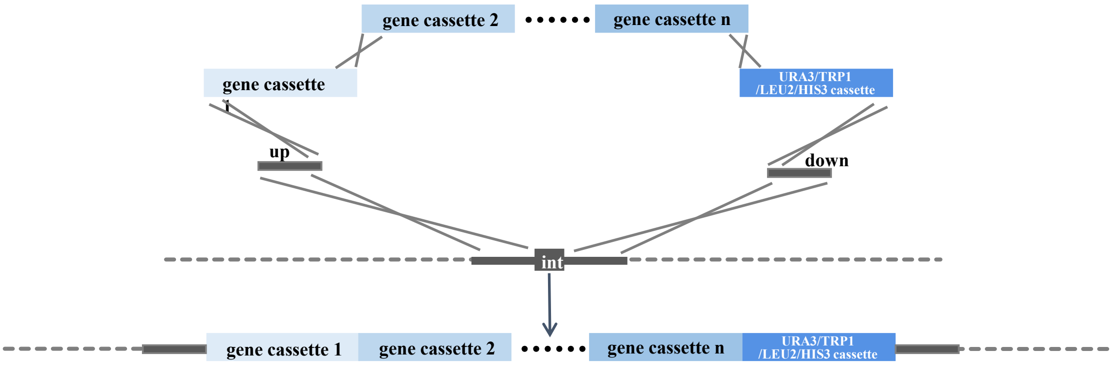

Figure S1 Targeted integration of multiple gene cassettes.

Up/down, up- and downstream flanks of integration site (int).

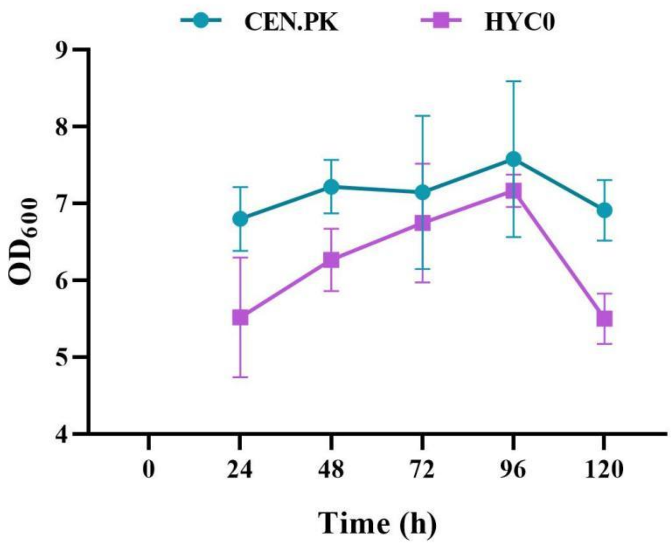

Figure S2 Cell growth status of CEN.PK and HYC0.

Samples were taken at 24 h, 48 h, 72 h, 96 h and 120 h during shake flask fermentation.

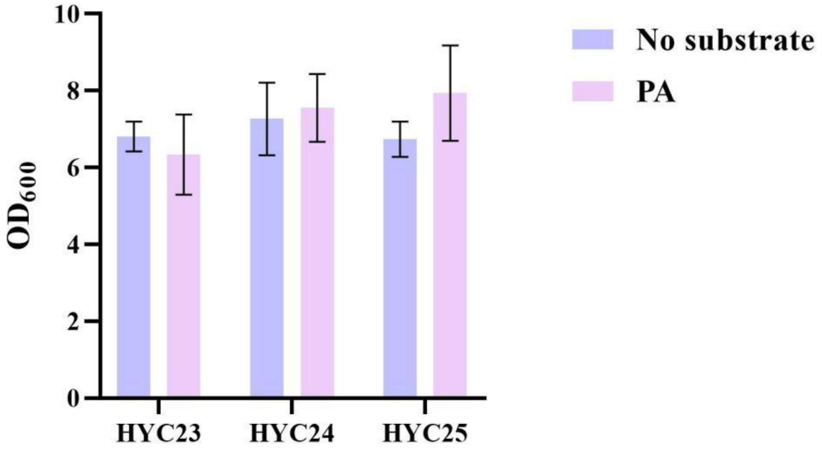

Figure S3 OD$_{600}$ of strain HYC23-25.

"PA" represents Shake flask fermentation results of HYC23, HYC24, and HYC25 by addition of PA and glycerol. "No substrate" indicates Shake flask fermentation results without addition of PA or glycerol. Samples were measured after 72 h.

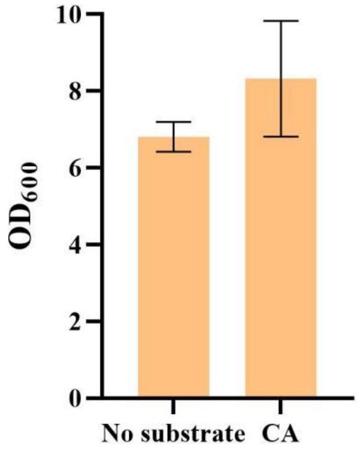

Figure S4 OD$_{600}$ of strain HYC23.

"CA" represents Shake flask fermentation results of HYC23 by addition of CA and glycerol. "No substrate" indicates Shake flask fermentation results without addition of CA or glycerol. Samples were measured after 72 h.

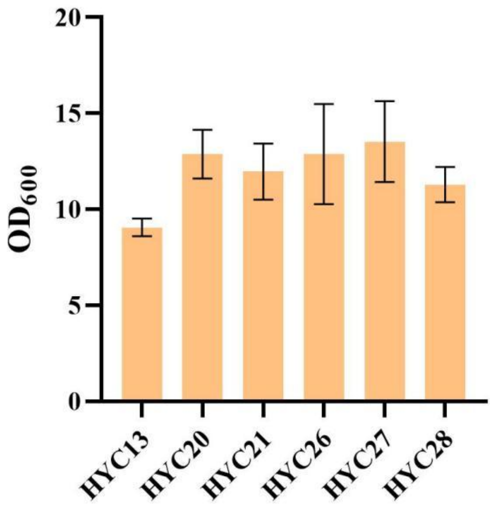

Figure S5 Cell growth status of HYC13, HYC20, HYC21, HYC26, HYC27, and HYC28.

The OD_{600} values were measured after 120 h.

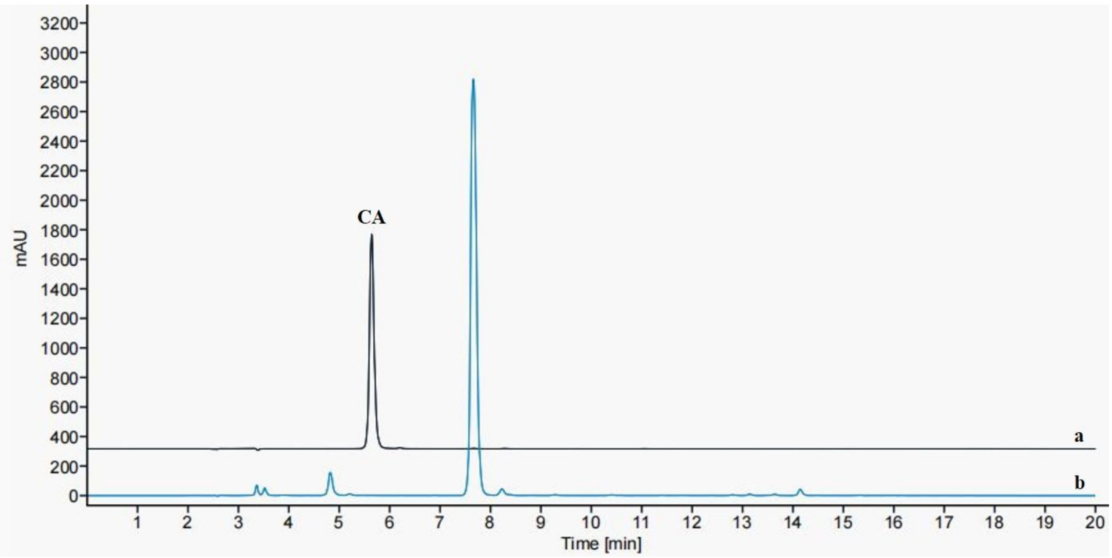

Figure S6 Typical HPLC profile of products in yeast strain HYC 21.

Line a: CA standard. Line b: Products in HYC21.

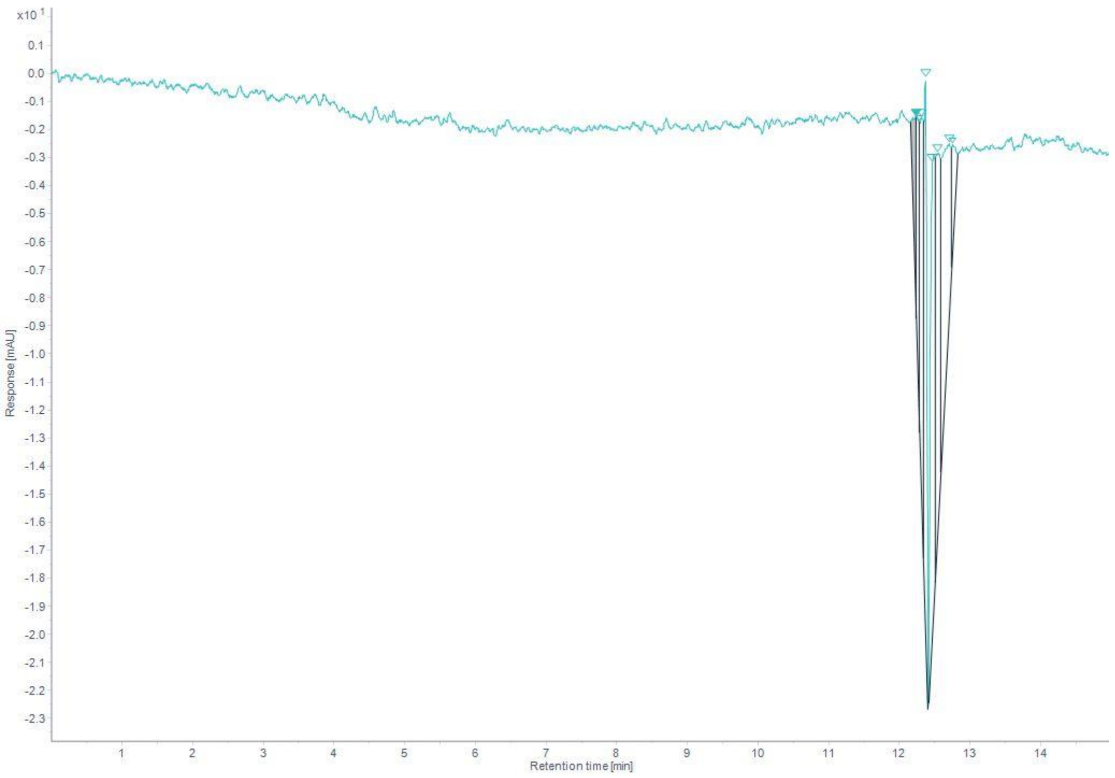

Figure S7 HPLC profile of PA-CoA standard.

The PA-CoA standard reference was purchased from Beiwanta Co. Ltd (Shanghai, China). The HPLC conditions for determining PA-CoA followed the manufacture's instructions, with the exception of using a reverse-phase C18 column. The gradient mobile phase was composed of Solvent A (0.2% trifluoroacetic acid in water) and Solvent B (acetonitrile) and was applied as follows: 0–8 min, 80% A and 20% B to 0% A and 100% B; 8–9 min, 0% A and 100% B to 0% A and 100% B; 9–10 min, 0% A and 100% B to 80% A and 20% B; 10–15 min, 80% A and 20% B to 80% A and 20% B. The flow rate was set at 1 mL/min, and PA-CoA was detected at 333 nm.

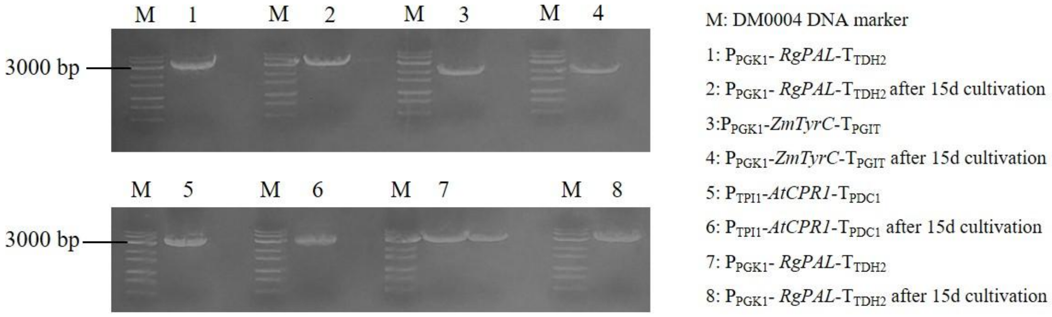

Figure S8 Genetic stability analysis of HYC13 and HYC20.

1-6: HYC20; 7,8: HYC13
